# Shotgun proteomics, *in-silico* evaluation and immunoblotting assays for allergenicity assessment of lesser mealworm, black soldier fly and their protein hydrolysates

**DOI:** 10.1038/s41598-020-57863-5

**Published:** 2020-01-27

**Authors:** Giulia Leni, Tullia Tedeschi, Andrea Faccini, Federico Pratesi, Claudia Folli, Ilaria Puxeddu, Paola Migliorini, Natasja Gianotten, Johan Jacobs, Stefaan Depraetere, Augusta Caligiani, Stefano Sforza

**Affiliations:** 1 Department of Food and Drug, University of Parma, Parma, Italy; 2 Centro Interdipartimentale Misure “Giuseppe Casnati”, Parma, Italy; 3 Department of Clinical and Experimental Medicine, University of Pisa, Pisa, Italy; 4 Protifarm, Ermelo, The Netherlands; 5 Circular Organics, Turnhout, Belgium

**Keywords:** Assay systems, Proteomics

## Abstract

Since 2018, insects have belonged the category of Novel Foods and the presence of allergens represents one of the main hazards connected to their consumption, also due to the potential cross-reactivity with Arthropoda pan-allergens. In the present work, the allergenicity assessment of black soldier fly and lesser mealworm was performed with a shotgun bottom-up proteomic approach combined with *in-silico* assessment, followed by IgG- and IgE-immunoblotting experiments. The peptides identified, filtered for their abundance and robustness, belonged mainly to muscle proteins, which represented the most abundant protein group. The relevant potential allergens were *in-silico* identified by sequence similarity to known allergens, and among them tropomyosin resulted the most abundant insect allergen. IgG-immunoblotting analysis with anti-Tropomyosin I antibodies and IgE-immunoblotting assay with serum from patient allergic to crustacean tropomyosin were performed in order to assess the immunoreactivity in both insects. The immunoassays were carried out also on protein hydrolysates extracted by treating insects with Protease from *Bacillus licheniformis* (1%, 60 °C, pH 7.5). While IgG-immunoblotting demonstrated the loss of immunoreactivity for both hydrolysates, IgE-immunoblotting showed a partial immunoreactivity preservation, also after hydrolysis, in the case of black soldier fly hydrolysate, and a total loss of immunoreactivity for lesser mealworm hydrolysate

## Introduction

Novel food protein sources are being studied in order to meet the future requirement for food, in connection to the perspectives of growing population^1^. Insects represent a good source of proteins, not only because of the high protein content in some species, but also for their nutritional quality in terms of essential amino acids profile. Not of a secondary importance, insect breeding, in comparison to common livestock, is characterized by many environmental advantages, such as less land use, feed and water requirement, fewer greenhouse gas emission and high feed conversion ratio^2^.

From January 1^st^ of 2018, insects have been included in the category of novel foods and the European Food Safety Agency (EFSA) opinion is mandatory before their marketing^3^. In 2015, EFSA assessed for the first time the safety related to insect consumption. The potential hazards connected to the use of insects in food or feed were deemed to be related to exogenous and endogenous factors, which could be influenced also by harvesting and processing methods^4^. The presence of allergens represents one of the main endogenous risk related to the consumption of insects. Insect-based food ingredients could cause an allergenic response either due to a primary sensitization, or to a cross-reaction. Primary sensitization is related to the ability of insect proteins to elicit an allergic reaction not related to other food allergies. This risk is widespread mainly in regions where edible insects are commonly used (e.g. China)^5^. A cross reaction, more likely where insects are not commonly consumed, like western countries, is related to an IgE cross-reactivity between insect proteins and known allergens belonging to species taxonomically related (other Arthropoda such as mites and crustaceans)^6^. In the last five years the potential allergic risk of whole insects’ consumption has been studied and evaluated. Many authors demonstrated a cross-reactivity between insects and other Arthropoda (crustaceans, mite) identifying as pan-allergens different proteins involved in the muscle contraction (actin, tropomyosin, troponin C), in enzymatic pathway (arginine kinase, fructose diphosphate aldolase) or part of the hemolymphatic system (hexamerin 1B)^7–9^.

The texture and appearance of insects are perceived as strong barrier for future consumers, moving the industrial interest to processed insect (e.g. flour, extracted proteins)^10,11^. It is well known that technological processes may alter the allergenic properties of food proteins^12^, but in literature there are only few data about the impact of technological process on insect allergenicity. Broekman *et al*.^13^ and van Broekhoven *et al*.^14^ evaluated the effect of different thermal treatment (e.g. blanching, baking, frying, microwave heating) on mealworm immunoreactivity and concluded that heat processing did not lower the allergenicity of mealworm proteins but affected their solubility properties. In contrast, Pali-Schöll *et al*.^15^ demonstrated the ability of thermal treatment to reduce the potential allergic risk of *Locusta migratoria*. This difference could be due to the diverse combination time/temperature applied in the process and the different insect species tested. Pali-Schöll demonstrated also the ability of enzymatic hydrolysis to reduce insect immunoreactivity.

Enzymatic hydrolysis is indeed widely used in the food/feed sector in order to extract proteins from vegetables and meat by-products and is exploited also to obtain ingredients with bio and techno functional properties^16^. In our previous work, we have explored this biotechnological tool on insects, deeply investigating the composition of protein hydrolysates obtained^17^. The protease assisted extraction represents also an effective approach to reduce the allergenicity of different food matrices^18–20^ and was demonstrated useful also for crickets, mealworms and locust species^14,15,21^.

In the present study, for the first time the proteome of Lesser Mealworm (*Alphitobius diaperinus*, LM) and Black Soldier Fly (*Hermetia Illucens*, BSF) larvae was characterized by a shotgun proteomic approach, evaluated in order to find potential allergens, and to estimate their abundance, via *in-silico* assessment and *in vitro* immunoassays. Furthermore, the enzymatic hydrolysis was explored for these two insects as a possible way to reduce the allergenic risk related to the consumption of insect proteins.

## Results

### Shotgun characterization of insect proteome

**Figure 1:**
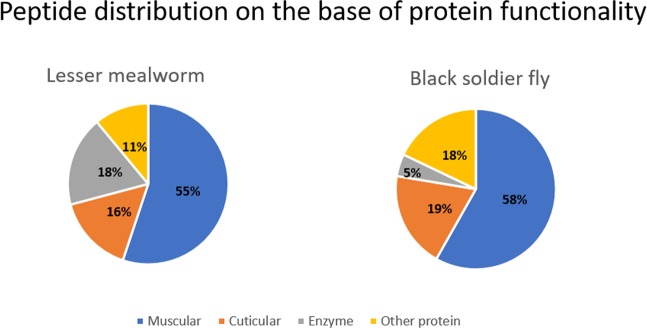
Distribution of peptides identified in LM and BSF protein extracts based on their functionality: muscular, cuticular, enzyme and other protein.

A shotgun proteomic approach was applied in order to evaluate the major determinants of the proteome of LM and BSF by High Resolution Mass Spectrometry (HRMS) on LTQ-Orbitrap instrument. Peptide identification was achieved by comparing the tandem mass spectra, derived from peptide fragmentation, with theoretical tandem mass spectra generated from *in-silico* digestion of *Insecta* protein database. The use of this targeted database, which only comprises insect proteins, increased the sensitivity of protein identification. A total of 261 and 107 peptides were identified, respectively in LM and BSF protein extracts. In order to reduce the presence of false positive, a data filtering was performed and the cut off arbitrarily set at 20 (−10lgP parameters in the PEAKS software® measuring the statistical significance of peptide-spectrum match) for the score and at ±6 ppm for mass accuracy. After data filtering, 127 and 67 peptides for LM and BSF, which were respectively mapped to 20 and 17 proteins, were retained and reported in detail in the Tables S1 and S2 in the Supplementary Material. Indeed, the application of such restricted parameters reduced the amount of identifiable peptides, but also allowed to focus our characterization on the more confident hits and most abundant proteins. In Fig. 1 we reported, with a schematic representation, the peptide distribution according to their functionality.

The main proteins identified by HRMS, both for BSF and LM, were muscle proteins (in particular actin, tropomyosin, myosin, troponin), which represented more than the 50% of identified proteins, followed by cuticular and metabolic proteins (enzymes and other proteins). It is important to underline that the *Insecta* database, used for the identification, is not complete, which implies a lesser amount of identified proteins, also in consideration to the strict cut off applied. In the Table 1 is reported a list of all the proteins identified, the number of peptides which covered the sequence and the peptide average Area. This last parameter was used to order the protein list according to their abundance, from the most abundant to the least abundant.

**Table 1:** The main proteins identified in both insects, Lesser mealworm and Black soldier fly, with information about the number of peptides, the average abundance and the protein functionality.

Lesser mealworm				Black soldier fly			
Protein	n° peptides	average Area	Function	Protein	n° peptides	average Area	Function
Actin	8	1.07E + 06	Muscular	Actin	10	7.13E + 04	Muscular
Myosin	39	5.67E + 05	Muscular	Cuticle protein	12	6.14E + 04	Cuticular
ADFb like protein	2	5.56E + 05	Other	Hexamerin	2	3.86E + 04	Other
Arginine kinase	5	5.28E + 05	Enzymatic	Tropomyosin	20	3.68E + 04	Muscular
Cuticle protein	18	3.99E + 05	Cuticular	Ca binding	2	2.69E + 04	Other
Tropomyosin	11	3.41E + 05	Muscular	Troponin	2	2.55E + 04	Muscular
Apolipophorin protein	2	3.40E + 05	Other				
Larval serum protein	6	3.15E + 05	Other				
ATP synthase	11	2.71E + 05	Enzymatic				
Paramyosin	5	2.71E + 05	Muscular				
Troponin	5	2.45E + 05	Muscular				
Chitin binding	2	2.12E + 05	Cuticular				
Catalase	2	7.51E + 04	Enzymatic				

We reported only the proteins represented by more than 2 peptides and isoforms of the same protein were grouped together under the parental protein.

For both LM and BSF, peptides from actin presented the highest average Area, clearly representing the most abundant protein. Actin is a multifunctional protein which express its function after the creation of a microfilament with other proteins. Actin is involved, with myosin, tropomyosin and troponin, in the formation of muscular myofibrils, engaged in the muscle contraction^7^. Myosin represented the protein with the highest amount of identified peptides in LM (39 peptides), whereas tropomyosin was the one having most peptides identified in BSF (20 peptides). Both proteins belong to the category of muscle proteins. All the above results demonstrate how the group of muscle proteins were the most abundant proteins, with the highest number of peptides identified (Fig. 1). Cuticle proteins, in both insects, were also detected with a high number of peptides, 18 peptides in LM and 12 in BSF. These proteins characterize the insect external coating and create a complex with chitin, which is the main constituent of the exoskeleton^22^.

In literature, especially in the last years, many authors have explored the proteome of edible insects, but only few of them have applied a shotgun approach. Rabani *et al*.^23^ evaluated the protein profile of BSF and blow fly (*Lucilia serenica*) for their future non-food applications. They performed a protein extraction by the trichloroacetic acid and acetone method, followed by tryptic digestion before the mass spectrometry analysis (by NanoLC-Ultra). Compared to the present work, they were able to identify a higher number of proteins, possibly due to the different extraction protocol and to the less strict criteria used for data filtering. Nevertheless, our method allowed highlighting those muscle proteins, which in Rabani *et al*.^23^ represented only a small proportion of the total, are indeed the most abundant. The protein patterns here determined were more similar to the one described by Yi *et al*.^24^ and Barre *et al*.^9^ for Yellow mealworm (*Tenebrio molitor*). Yi and colleagues applied for protein extraction a Filter Aided Sample Preparation (FASP) (involving the use of SDS, Urea and DTT). Barre and colleagues extracted the Yellow mealworm proteome in a tris-buffered saline solution and performed the mass spectrometry analysis with an LTQ-Orbitrap instrument. Furthermore, they also studied the distribution of detected proteins, according to their functionality, by identifying mainly enzymatic and functional proteins (65% of total protein identified). The difference in functional protein distribution with the present work could be related to the different species, and to the strict data filtering here applied, which focused on the most robust results and hence on the most abundant proteins.

By our knowledge, the protein profile of LM was described for the first time and also compared with the BSF pattern. This detailed protein identification constitutes the basis for the *in-silico* assessment of cross reactivity with known allergens.

### *In-silico* allergenicity assessment by Allermatch^TM^ tool

Bioinformatics tools are used to compare the amino acids sequence of a protein with the sequences of known allergens in order to determine sequence similarity. Based on the results of this alignment it is possible to discover the presence of potential allergens. In fact, FAO/WHO 2001 and Codex Alimentarius 2003 reported that 35% sequence identity to known allergen over a window of at least 80 AA is considered a minimal requirement to regard a protein allergenic in nature^25^.

In the present work, we decided to focus our attention on the peptide sequences actually identified and not to the potential parental protein from which they occur, in order to avoid a less robust allergenicity assessment, due to the incomplete *Insecta* database. The identified peptides were matched with allergen sequences using Allermatch^tm^ tool and we obtained a positive hit for 32 peptides from LM and for 25 peptides from BSF. In order to avoid false positive results, we performed a data filtering considering only peptides with more than the 50% of wordmatches (Table 2).

**Table 2:** Identified peptides which presented a positive hit with known allergens.

Peptide	Protein	% wordmatches	Species	Allergen	Class
**Lesser mealworm**					
LIDDHFLF	Arginine kinase	100	*B. germanica*	Bla g 9	Insect
IVELEEELR	Tropomyosin	100	*M. rosenbergii*	Mac r 1	Prawn
MYDGIAELIK	Arginine kinase	100	*B. mori*	Bomb m 1	Insect
YKEIGDDLD	Tropomyosin	100	*C. kiiensis*	Chi k 10	Insect
VIQSGLENHDSGIGIYAPDAD	Arginine kinase	56	*B. germanica*	Bla g 9	Insect
FLAEEADKKYDEVAR	Tropomyosin	100	*C. kiiensis*	Chi k 10	Insect
LQLIEEDLER	Tropomyosin	80	*L. destructor*	Lep d 10	Mite
IQLLEEDLER	Tropomyosin	100	*M. rosenbergii*	Mac r 1	Prawn
IMELEEELK	Tropomyosin	100	*L. saccharina*	Lep s 1	Insect
MDALENQLK	Tropomyosin	100	*M. rosenbergii*	Mac r 1	Prawn
VSSTLSGLEGELK	Arginine kinase	100	*B. germanica*	Bla g 9	Insect
LEEVASKF	Arginine kinase	67	*B. mori*	Bomb m 1	Insect
QLQEQEGMSQQNVTR	House dust mite allergen	50	*D. pteronyssinus*	Der p 11	Mite
LAEASQAADESER	Tropomyosin	100	*M. rosenbergii*	Mac r 1	Prawn
STAGDTHLGGEDFDNR	Heat shock protein 70	100	*D. farinae*	Der f 28	Mite
NALEQANKDLEEKEK	Myosin	80	*A. aegypti*	Aed a 10	Insecta
**Black soldier fly**					
DRLEDELGLNK	Tropomyosin	50	*L. saccharina*	Lep s 1	Insect
DRLEDELGINK	Tropomyosin	100	*L. saccharina*	Lep s 2	Insect
IVELEEELR	Tropomyosin	100	*M. rosenbergii*	Mac r 1	Prawn
IQLLEEDLER	Tropomyosin	100	*M.rosenbergii*	Mac r 1	Prawn
IMELEEELK	Tropomyosin	100	*L. saccharina*	Lep s 2	Insect
MDALENQLK	Tropomyosin	100	*M. rosenbergii*	Mac r 1	Prawn
MDQLTNQLK	Tropomyosin	100	*L. saccharina*	Lep s 2	Insect
MVEADLER	Tropomyosin	100	*M. rosenbergii*	Mac r 1	Prawn
LAFVEDELEVAEDR	Tropomyosin	100	*A. aegypti*	Aed a 10	Insect
LLAEDADGK	Tropomyosin	75	*L. saccharina*	Lep s 1	Insect
LSEASQAADESER	Tropomyosin	75	*M. rosenbergii*	Mac r 1	Prawn
FRAAVPSGASTGVHEALELR	Beta-enolase	100	*S. salar*	Sal s 2.0101	Fish
LAMVEADLER	Tropomyosin	100	*M. rosenbergii*	Mac r 1	Prawn
QLIEEDLER	Tropomyosin	100	*L. destructor*	Lep d 10	Mite
QLLEEDLER	Tropomyosin	100	*M. rosenbergii*	Lep d 10	Prawn
LEVSEEK	Tropomyosin	100	*M. rosenbergii*	No name	Prawn
ALGFPFDR	Haemolymph	66.67	*B. germanica*	Per a 3	Insect
SLEVSEEK	Tropomyosin	100	*M. rosenbergii*	No name	Prawn

The *in-silico* assessment was carried out with Allermatch^TM^ on both lesser mealworm and black soldier fly peptides identified. After data filtering, we reported only the results which presented more than the 50% of wordmatches.

After data filtering, we identified positive hits for 16 peptides from LM and 18 from BSF, corresponding respectively to the 13% and 27% of total peptides identified. The relevant allergens detected after alignment belong mainly to two distinct classes, associated to two distinct animal groups: allergens belonging to crustaceans, so very relevant for allergic response after ingestion as food, or allergens belonging to insects already known as being responsible for inhalatory allergies or after stinging (other insects and mites). Furthermore, the identified peptides from LM and BSF showed high sequence identity with tropomyosin and arginine kinase, well known allergens for both classes^26,27^. The 73% of potential allergenic peptides presented a complete word match (100%) with the amino acids sequence of known allergens. This indicates not only a close similarity of this protein regions between species taxonomically related, but also the risk of cross-reactivity for persons allergic to crustaceans and house dust mites.

Between the two potential allergens identified, tropomyosin resulted the most impacting due to the high similarity with other known tropomyosin allergens. In fact, tropomyosin from shellfish, house dust mite and insects (American cockroach) share a high degree of similarity, round 75–80%^8^. This similarity raises the possibility of reactivity to shellfish tropomyosin due to sensitization from inhalatory insect sources. The allergenicity risk for both insects could be related to ingestion and inhalation. Given the inhalator aspect, this risk is to be considered not only for potential consumers, but also for workers having to deal with insect rearing and fractionations.

Thus, all the above data hints at tropomyosin as the potential prevalent allergen in eventual allergic reactions due to insect consumption.

### IgG- and IgE-immunoblotting experiments

In order to deeply explore the allergenic potential of tropomyosin from LM and BSF we performed IgG- and IgE-immunoblotting analysis. For IgG-immunoblotting, the assay was performed using anti-tropomyosin I (TPM1) antibody, produced from the 92–273 AA sequence of human Tropomyosin. This antibody has been chosen since it has been obtained against an amino acid sequence that includes immunogenic regions highly conserved in all tropomyosin forms. Thus, in this way we maximized the chances to recognize, with this antibody, also insect tropomyosin, whatever form was present. As a matter of fact, scarce information is present in literature about how many forms of tropomyosin come from alternative splicing. The presence of alternative isoforms might also justify the presence of several bands of various dimensions in the blotting analyses.

**Figure 2:**
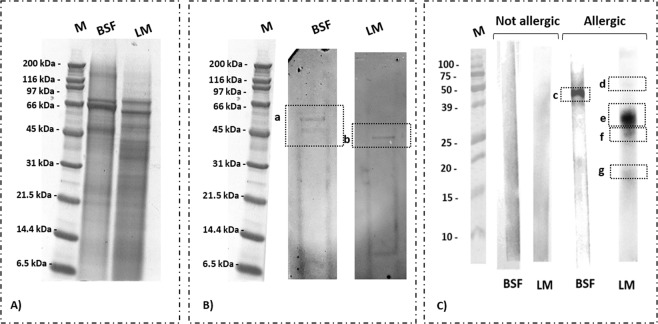
(**A**) SDS-Page of: black soldier fly (BSF) and lesser mealworm (LM) larvae; (**B**) IgG-immunoblotting of the samples separated by SDS-Page, followed by incubation with anti-tropomyosin IgG; (**C**) IgE-immunoblotting results after incubation with sera from non-allergic patient and person allergic to crustacean tropomyosin. Response from IgG binding can be visible in a and b, while from IgE binding in **c**–**g**. M: marker. Full-length blots/gels are presented in Supplementary Figs. S1–S3.

The protein profile of LM and BSF presented some similarity (Fig. 2A). In particular, major bands were visible in both lines: one band at 45 kDa and two at 66 kDa, which could be related respectively to myosin, hexamerin and cuticle protein. Under 45 kDa other bands described the protein pattern of both insects, especially for LM. The band round 31 kDa could be related to tropomyosin, even if the presence of a lot of isoforms made the correct association difficult^28^. The IgG-immunoblotting scan are reported in Fig. 2B. Anti-TMP1 antibody was able to bind a protein in the extract from raw material (LM and BSF lines). In LM extracts the TMP1 antibody was able to bind a protein with a MW between 45 and 31 kDa, whereas in BSF the antibody was able to bind two proteins in the range between 66 and 45 kDa.

In order to really demonstrate that TPM may actually be relevant in allergy, we performed an immunoassay with serum from allergic person to crustacean tropomyosin (Pen a 1) (Fig. 2C). In both insects we identified the presence of reactivity with IgE from serum of allergic patient, while in the control (person without allergy) no reactivity was determined. In the BSF profile a strong reactivity was determined between 50 and 39 kDa, while in the LM profile between 39 and 25 kDa, which matched with the ones detected in the IgG-immunoblotting assays. For LM, we identified other two minor reactivity at 50 kDa and between 20 and 15 kDa.

The above data definitely confirmed the potential allergenicity of insect tropomyosin, also in cross reactivity with crustacean allergens.

### Influence of enzymatic hydrolysis on allergenic properties

The allergenic risk of insects represents a critical point for their future consumption and for this purpose we explored the enzymatic hydrolysis as a biotechnology tool for decreasing the allergenic potential of both LM and BSF. The enzyme employed was the Protease from *Bacillus licheniformis*, which was used as a technological adjuvant not only to extract the protein fraction, but at the same time also to reduce the protein molecular size. Van Broekhoven *et al*.^14^ demonstrated the ability of gastro-intestinal enzymes to reduce the immunoreactivity of LM, while Hall *et al*.^21^ and Pali- Schöll *et al*.^15^ obtained the same results but by processing different insects with Alcalase. In the present work, for the first time, BSF was subjected to enzymatic hydrolysis and the potential reduction in immunoreactivity evaluated.

**Figure 3:**
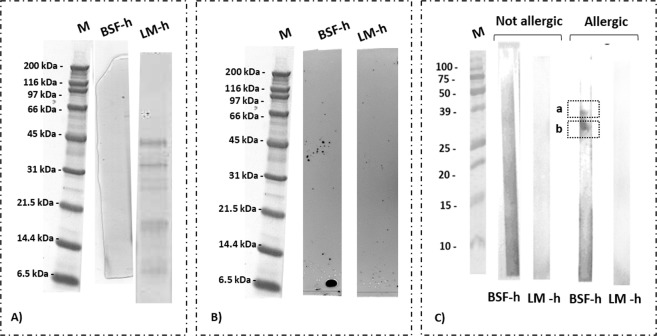
(**A**) SDS-Page of: protein hydrolysates obtained from proteolysis activity of the protease from *Bacillus licheniformis* on black soldier fly (BSF-h) and lesser mealworm (LM-h) protein hydrolysates; (**B**) IgG-immunoblotting of the samples separated by SDS-Page, followed by incubation with anti-tropomyosin I IgG; (**C**) IgE-immunoblotting results after incubation with sera from non-allergic patient and person allergic to crustacean tropomyosin. Response from IgE binding in a and b. M: marker. Full-length blots/gels are presented in Supplementary Figs. S4–S8.

The enzymatic hydrolysis was performed as described in Leni *et al*.^17^. The hydrolysates were rich in protein compounds, since 79 ± 7% and 70 ± 1% of total proteins were extracted respectively from BSF and LM. The protein fractions obtained were mainly composed of peptides and the degree of hydrolysis, calculated by OPA assay, was 10.4 ± 2.3% for BSF and 21.8 ± 0.5% for LM. From the reverse of these values it was possible to theoretically calculate the average peptide length, which resulted in 8 to 12 amino acid residues for BSF and an average peptide length of 4 to 5 for LM. As described by Nagodawithana *et al*.^29^ the peptide length directly influences the allergenicity properties of a protein hydrolysates, demonstrating that an average molecular weight lower than 1500 Da can reduce the allergenicity property of a food product. Assuming an average molecular mass for residual amino acid of 110 Da, this means that the hydrolysates here could potentially be defined as hypoallergenic, even if the following results from IgE-immunoblottig (Fig. 3C) revealed the presence of immunoreactivity in BSF hydrolysate, outlining the presence of residual intact proteins. Indeed, as reported by a European Union directive, the hypoallergenicity has to be assessed by showing the absence of orally sensitisation after animal administration^30^, but certainly the possibility to degrade the allergenic proteins to small peptides might help to achieve this result.

In Fig. 3A we show the protein profile of LM hydrolysate, where three major bands are visible round 45 kDa, 21.5 kDa and 14.4 kDa. No proteins were displayed at molecular weight higher than 45 kDa, which were present in the whole LM pattern instead. In BSF hydrolysate no net bands were identified in the gel.

IgG-immunoblotting assay was performed also on these protein hydrolysates, and the results are reported in Fig. 3B. In both LM and BSF hydrolysates, no reactivity with the anti-TPM1 antibody was identified. The enzymatic hydrolysis apparently eliminated the antibody ability to bind the target sequence in tropomyosin. This result was in line with what reported by Pali- Schöll *et al*.^15^. Hall *et al*.^21^ and van Broekhoven *et al*.^14^, respectively for locust, crickets and mealworms, where after enzymatic hydrolysis there was a visible reduction of reactivity between insect proteins and the antibodies in crustaceans and house dust mite allergic patients. The absence of reactivity was also underlined in the IgE-immunoblotting assay of the protein hydrolysate obtained from LM, where the reactivity determined in the protein extract here was not evident (Fig. 3C). On the contrary, the BSF protein hydrolysate kept an evident reactivity with the IgE from allergic patient between 39 and 25 kDa, which could be due to fragments released during the enzymatic hydrolysis from the intact protein, but still retaining the ability to be recognized by IgE antibodies(Fig. 2C). The enzymatic assisted extraction, as here performed on BSF, was then not sufficient to completely eliminate the cross-reactivity between insect tropomyosin and the human IgE.

In general, the enzymatic assisted extraction here presented could be a good strategy to obtain hypoallergenic extracts not only from LM, as already reported in literature^13^, but also from BSF, even if its real efficacy in all cases will have to be carefully further studied and optimized in order to achieve a sufficient DH able to completely suppress IgE reactivity.

In particular, the less efficient reactivity of the enzyme and the variability of the results can be ascribed to the fact that the hydrolyzates have been here obtained by ground insects, not by soluble homogeneous protein extracts. Inhomogeneous solid material is hydrolyzed much less efficiently than soluble proteins, and other factors, beside protein susceptibility, play an important role (such as chitin or lipid presence).

## Conclusion

In the present work the allergenicity assessment of two common edible insects, LM and BSF, was evaluated by both proteomics and *in vitro* assay, and the enzymatic hydrolysis performed as a biotechnological tool to produce hypoallergenic fractions from these two insect species.

Major proteins in the proteome of both insects were determined with a shotgun proteomic approach by HRMS on LTQ-Orbitrap instrument, followed by a severe data filtering of the identified peptides in order to focus on the most abundant and the most certainly identified insect proteins. The peptides identified mainly belong to the category of muscle proteins, with actin, tropomyosin and myosin representing the most abundant ones. The proteomic characterization posed the basis for the subsequent *in-silico* allergenicity assessment with the Allermatch^TM^ tool, which evaluated the presence of similarity between the identified peptides and the sequence of known allergens. Tropomyosin was identified as the major pan-allergen, underling a strong similarity with other tropomyosin from crustaceans and dust mites, known to be responsible of food and inhalatory allergy. An IgG-immunoblotting analysis conducted by using antibodies directed against tropomyosin I, recognized specific bands in extracts from both insects. An IgE-immunoblotting experiment, performed with serum of patient allergic to crustacean tropomyosin, then confirmed in a more allergen-relevant environment, the potential of tropomyosin to be the prevalent insect allergen.

In view of the future expected use of insects as food sources, we explored the enzymatic hydrolysis as a way to reduce the allergenic properties of LM and BSF. For BSF, this approach was explored for the first time in the present work. The protein hydrolysates demonstrated complete disappearance of immunogenic reactivity in IgG-immunoblotting experiments, and complete disappearance in LM and partial reduction in BSF of immunogenic reactivity in IgE-immunoblotting analysis. In particular, in BSF protein hydrolysate we identified two protein fragments keeping the immunoreactivity.

In conclusion, the data here presented confirms the allergenic potential of insect proteins. For the first time a proteomic approach clearly indicated tropomyosin as the prevalent potential allergen. The cross-reactivity for the subjects already allergic to crustacean tropomyosin was clearly proven by immunoblotting experiments. Enzymatic hydrolysis was confirmed as effective strategy to reduce LM allergenic risk and demonstrated potentially valid also for BSF, even if deeper investigations are needed in order to adjust the condition of hydrolysis, achieving a sufficient DH to obtain effective hypoallergenic properties.

The results obtained are relevant since they indicate different immunoreactivity can still remain in different species, even when subjected to the same enzymatic hydrolysis. We also demonstrated clearly that the simple measure of DH is not enough to assess hypoallergenicity and that IgE reactivity and possibly *in vivo* challenges are needed.

## Material and Methods

### Insect sample

BSF larvae were provided by Circular Organics (Turnhout, Belgium), whereas LM larvae by Protifarm (Ermelo, The Netherlands). BSF and LM larvae were reared as described in Leni *et al*.^17^. Larvae were killed by packing them vacuum sealed and freezing at −30 °C. After one week, samples were freeze-dried and stored at −20 °C for the future analysis. Samples were grinded for 2 minutes with IKA A10 laboratory grinder (IKA Werke GmbH & Co. KG, Germany) before each analysis.

### Protein extraction

Grinded insects were defatted with diethyl ether following the method proposed by Caligiani *et al*.^31^ using a Soxhlet extractor (SER 148/3 VELP SCIENTIFICA, Usmate Velate, Italy). Defatted flours were subjected to the protein extraction protocol proposed by Abdel Rahman *et al*.^32^ with some modifications. Briefly, 0.5 g of sample were homogenized in an ice-bath with 10 mL of a Tris buffer (25 mM Tris-HCl pH 8.0, 1 M KCl, 50 mM DTT and 0.5 mM EDTA) using Ultra Turrax homogenizer (11000 rpm) for 1 minute. The mixture was left in an ice bath under agitation for 7 hours. The slurry was than centrifuged at 2683 g for 30 minutes at 4 °C and the supernatants collected were subjected to desalting using 3 kDa Amicon® Ultra Centrifugal Filters (Merck Millipore). The final protein concentration was determined using the Qubit Protein Assay Kit and the Qubit 2.0 fluorometer (Invitrogen, California, USA). The protein extraction was performed in duplicate and the extracts were used for further protein molecular characterization and allergenicity assessment.

### In-solution tryptic digestion

The protein identification was carried out with a Shotgun proteomic approach following the “In-solution tryptic digestion” method proposed by Kinter *et al*.^33^. In particular, the defatted grinded insect larvae were suspended in 6 M Urea, 100 mM Tris-HCl pH 7.8 at a final protein concentration of 10 mg/mL. The mixture was homogenized at 4 °C and centrifuged at 2683 g for 30 minutes at 4 °C. 100 µL of the supernatant was mixed by gentle vortex with 5 µL of a reducing solution (200 mM DTT, 100 mM Tris-HCl pH 7.8) in order to reduce the disulfide bonds. After 1 hour, 20 µL of an alkylation reagent (200 mM iodoacetamide, 100 mM Tris-HCl pH 7.8) was added and mixed by gentle vortex. After 1 hour, 20 µL of the reducing solution was added to consume any unreacted iodoacetamide and mixed by gentle vortex. After 1 hour, the urea concentration was reduced by diluting the mixture with 775 µL of MilliQ® water and combined with 100 µL of a trypsin solution (200 ng/µL trypsin and 100 mM Tris-HCl pH 7.8). The samples were mixed with gentle vortex and the trypsin digestion carried out overnight at 37 °C. The reaction was stopped decreasing the pH below 6 by adding acetic acid. The digests were dried under nitrogen before the mass spectrometry analysis.

#### Protein identification by high resolution mass spectrometry on LTQ-orbitrap instrument

The dry protein extracts were reconstituted with 50 μL of 0.2% formic acid solution for mass spectrometric analysis. High resolution mass spectrometry was performed on the samples for peptide identification using a μHPLC DIONEX Ultimate3000 interfaced with a LTQ-Orbitrap XL Thermo Fisher Scientific. Column: Jupiter C18 4 μ, Proteo 90 Å 150 × 0.30 mm, Phenomenex; eluent A: water + 0.1% formic acid; eluent B: acetonitrile + 0.1% formic acid; flow: 5 μL/min, gradient: 0–4 min from 100% A to 95% A, 4–60 min from 95% A to 50% A, 60–62 min from 50% A to 10% A, 62–72 min 10% A, 72–74 min from 10% A to 95% A, 74–90 min 95% A; analysis time (min): 90; column temperature (°C): 30; injection volume (μL): 5; acquisition time (min): 0–75; ionization mode: ESI+; scan range (m/z): 200–1800; source voltage (kV): 3.5; capillary voltage (kV): 35; source temperature (°C): 275. Scan event details: (Fourier transform) FTMS + p res = 30,000 or (250.0–2000.0); (ion trap) ITMS + c Dep MS/MS Most intense ion form; activation type: CID; isolation width: 2.00; normalized coll. energy: 35.0; default charge state: 2; activation Q: 0.250; activation time: 30.000; dynamic exclusion enabled; repeat count: 2; repeat duration (s): 10.00; exclusion duration (s):30.00. Charge state rejection: enabled; unassigned charge states: rejected; charge state 1: rejected; charge state 2: not rejected; charge state 3: not rejected; charge states 4+: not rejected; ion signal threshold: 10,000. Protein identification was performed by using PEAKS software (Bioinformatics Solutions Inc) and INSECTA (UniProt) database. Positive hits for protein identification was arbitrarily set for all those proteins identified by the program with a score (expressed as −10lgP) > 50, all those peptides with a score (−10lgP) > 20 and ppm in the range ± 6, since such value should reduce the risk of false positives to zero.

#### *In-silico* analysis for the prediction of allergenicity

The UniProt database (www.uniprot.org) was employed to retrieve information about the sequences identified in BSF and LM protein extracts by HRMS. For the prediction of allergenicity we used Allermatch^TM^ tool (www.allermatch.org) by which the amino acid sequence of a protein of interest can be compared with sequences of allergenic proteins. Peptides identified, after data filtering, were entered in the web tool and analyzed with the word match method. Positive hits as potential allergens were arbitrarily taken for all the peptides having more than 50% of exact wordmatches with known allergens.

### Enzymatic assisted extraction

Protease from *Bacillus licheniformis* (≥2.4 U/g; EC Number 3.4.21.62) was used in order to produce a peptide rich fraction from grinded LM and BSF larvae. The hydrolysis reactions were carried out overnight on 5 g of ground insects, 45 mL of a phosphate buffer (Na_2_HPO_4_ 10 mM) and 1% of enzymes, and performed as described in Leni *et al*.^17^. The final hydrolysates were collected at −20 °C for subsequent analysis.

#### Degree of hydrolysis

The degree of hydrolysis (DH), which is defined as the percentage of cleaved peptide bonds in a protein hydrolysate, was calculated using o-phtaldialdehyde (OPA) method as described by Leni *et al*.^17^. The DH was calculated as the ratio between the free nitrogen groups after hydrolysis and the total nitrogen groups: DH% = (Nfree/Ntotal) × 100. The first value was calculated by the OPA reactivity. The total proteinaceous N of proteins was calculated by considering the total N and by separating the protein N contribution from the chitin one, as described for these two insect species in our previous work^17^.

### SDS–PAGE and IgG-immunoblotting

Sodium dodecyl sulphate polyacrylamide gel electrophoresis (SDS-PAGE) was used to determine the molecular weight distribution of the insect protein extracts and protein hydrolysate and performed as described in Leni *et al*.^34^. Protein fractions were analyzed on 12% Bis/Tris Criterion™ XT Bis-Tris Gel (Bio-Rad, Hercules, CA, U.S.A) by using MES running buffer. The gel was finally stained with Coomassie Brilliant Blue, destained and finally scanned with GS-800 Calibrated Densitometer controlled by the software “Quantity one” (BIO-RAD). IgG-immunoblotting was performed on the same samples used for SDS-PAGE, which were separated on gels and electro-transferred onto nitrocellulose membranes. The assays were performed using anti-TPM1 Polyclonal Antibody (Thermo Fisher PA5-29846) produced against the highly conserved 92–273 AA sequence of human tropomyosin. The membrane was incubated under agitation overnight with 5% skimmed milk powder in incubation buffer TT (0.3% Tween 20 in TBS pH 9.6). The blot was washed three times using the TT buffer and then incubated with primary antibody anti-TPM1 polyclonal Antibody (1:250 dilution in TT buffer) at room temperature for 1 hour under agitation. The blot was washed three times with TT buffer and then incubated with Rabbit IgG Secondary Antibody (polyclonal) conjugated with DyLight 680 (1:5000 dilution in TT buffer) for 1 hour at room temperature under agitation. The membrane was washed extensively with TT buffer and then preserved in 50 mM Tris pH 7.5 until the detection with Li-Cor Odyssey Infrared Imaging System.

### IgE-immunoblotting

Twenty μL of insect protein extracts and hydrolysate were analyzed using 15% acrylamide/Tris–HCl gels (Criterion, Biorad, Germany). After electrophoretic run, proteins were transferred to a polyvinylidene difluoride membrane (Bio-Rad). Membranes were blocked with 5% (w/v) of BSA in incubation TT buffer (0.05% Tween in TBS) for 60 min after which the membrane was incubated overnight at 4 °C with diluted sera (1:10 in TT, BSA 5%) from non-allergic person and patient who showed a food allergy sensitisation to crustacean, as determined by skin test, and, in particular, with IgE positivity to tropomyosin (Pen a 1). After incubation the blot was washed three times with TT buffer and then incubated with Anti-human IgE diluted 1:10000 in TT buffer for 90 min. Bound IgE was detected using a chemiluminescent peroxidase substrate kit, blots were scanned using a BioRad Versadoc 1000 image scanner (Bio-Rad) and images analysed using Quantity One BioRad software.

## Supplementary information

Supplementary material.
