# Supplementary material for: Shotgun proteomics, in-silico evaluation and immunoblotting assays for allergenicity assessment of lesser mealworm, black soldier fly and their protein hydrolysates
Source: Sci Rep. 2020 Jan 27;10:1228. doi: 10.1038/s41598-020-57863-5 (PMC6985256; doi:10.1038/s41598-020-57863-5)
Supplement: Supplementary file 1 — Supplementary material. [file 41598_2020_57863_MOESM1_ESM.pdf]

Table S1 Peptide sequences identified in black soldier fly protein extract by High Resolution Mass Spectrometry on LTQ-Orbitrap instrument. The characterisation reported information about the precision of the MS identification (10lgP and ppm), the peptide abundance (Area), the Uniprot accession number and also the name of the parental protein.

Peptide	-10lgP	ppm	Area	Accession	Protein
NLGTDAEAIVVR	22.97	3.6	2.44E+05	B0XA27_CULQU	Larval cuticle protein 8.7
VADENGFQPEGAHIPK	30.21	2.5	1.98E+05	B0XA27_CULQU	Larval cuticle protein 8.8
VAPEEHPVLL	26.7	3.3	1.73E+05	A0A1B0CWE9_LUTLO	Uncharacterized protein (Actin ATP binding)
VDGFDLK	21.29	4.1	1.71E+05	A0A0M4EJM5_DROBS	Fringe glycosyltransferase
SFKDDFLEK	23.61	4.2	1.43E+05	A0A182SYB4_9DIPT	Uncharacterized protein (GNEFA)*
EITALAPSTIK	38.65	3.2	1.02E+05	A0A1B0CWE9_LUTLO	Uncharacterized protein (Actin ATP binding)
EITALAPSSIK	24.72	3.2	1.02E+05	F1C3P6_TIMCA	Actin
WGFETSDGK	22.85	3.1	9.65E+04	C0H6J6_BOMMO	Putative cuticle protein
DRLEDELGLNK	59.11	3.9	9.60E+04	E2A6N1_CAMFO	Tropomyosin-1
DRLEDELGINK	59.11	3.9	9.60E+04	A0A158NXC8_ATTCE	Uncharacterized protein (tropomyosin)
ALLPDGK	26.53	4.5	9.17E+04	E2C0X6_HARSA	NHL* repeat
IVELEEELR	44.82	2.9	6.68E+04	B4QYK2_DROSI	GD19006 (Tropomyosin)
AGFAGDDAPR	46.01	3.5	6.60E+04	A0A1A9ZNP6_GLOPL	Uncharacterized protein (Actin ATP binding)
VDLVEGK	24.06	3.8	6.60E+04	B0WYN8_CULQU	Dipeptidyl - peptidase
FPLDMSEAH	21.75	3.4	6.18E+04	U5EWU6_9DIPT	Putative hexamerin 2 beta
LADENGFQPEGAHLPR	22.46	4.1	6.13E+04	A0A1A9Z940_GLOPL	Uncharacterized protein (cuticle protein)
LEDELVIEK	37.64	3.7	5.78E+04	TPM_LOCMI	Tropomyosin
LEDELVLEK	37.64	3.7	5.78E+04	B0X3L6_CULQU	Tropomyosin invertebrate
DSYVGDEAQSK	53.04	3.8	5.59E+04	A0A1B0CWE9_LUTLO	Uncharacterized protein (Actin ATP binding)
GIITNWDDMEK	48.22	3.2	5.58E+04	A0A1B0CWE9_LUTLO	Uncharacterized protein (Actin ATP binding)
IQLLEEDLER	42.01	3.4	5.07E+04	B0X3L6_CULQU	Tropomyosin invertebrate
SGGTTMYPGIADR	26.13	4.5	5.01E+04	A0A1A9ZNP6_GLOPL	Uncharacterized protein (Actin ATP binding)
QEYDESGPGIVHR	45.35	3.2	4.95E+04	A0A1B0CWE9_LUTLO	Uncharacterized protein (Actin ATP binding)
SLEVSEEK	23.97	3.7	3.99E+04	A0A0Q9WML9_DROVI	Uncharacterized protein (tropomyosin isoform J)
LDLGLNK	22.68	4.5	3.95E+04	K7JVQ9_NASVI	Uncharacterized protein (Zinc ion binding)
GEGDPEFIK	28.33	3.7	3.94E+04	TNNT_DROME	Troponin T, skeletal muscle
IMELEEELK	31.51	3.1	3.64E+04	A0A1J1HZZ6_9DIPT	CLUMA_CG005729, isoform B (putative Tropomyosin 2)

LEYPELK	26.03	2.9	3.55E+04	A0A182IRM9_9DIPT	Uncharacterized protein (Ca binding)
MDALENQLK	42.36	4	3.21E+04	B0X3L6_CULQU	Tropomyosin invertebrate
VVDNGSGMCK	35.6	3.4	3.04E+04	A0A1B0CWE9_LUTLO	Uncharacterized protein (Actin ATP binding
SYELPDGQVITI	33.45	3.9	2.86E+04	A0A1A9ZNP6_GLOPL	Uncharacterized protein (Actin ATP binding
QLIEEDLER	30.89	5.3	2.79E+04	A0A158NXC8_ATTCE	Uncharacterized protein (Tropomyosin)
QLLEEDLER	30.67	5.3	2.79E+04	A0A182XV85_ANOST	Uncharacterized protein (Tropomyosin)
AYDVQDALTGDSK	46.87	3.3	2.77E+04	R4G8D1_RHOPR	Putative cuticle protein
VQQIEEDLEK	44.08	3.4	2.74E+04	A0A1L8EHE5_HAEIR	Putative tropomyosin-2 isoform x1
AYDVQDAITGDSK	42.58	4.7	2.61E+04	A0A026VY81_CERBI	Cuticle protein
GFETSDGK	22.89	4.3	2.19E+04	C0H6J6_BOMMO	Putative cuticle protein
MDQLTNQLK	28.79	3.5	2.04E+04	A0A1J1HX79_9DIPT	CLUMA_CG005729, isoform F (tropomyosin 2)
MVEADLER	32.92	3.4	1.84E+04	B0X3L6_CULQU	Tropomyosin invertebrate
TPDYLLR	28.53	3.6	1.83E+04	A0A1B6CX71_9HEMI	Uncharacterized protein (calcium ion binding)
TPDYILR	28.53	3.6	1.83E+04	B4LFD9_DROVI	Uncharacterized protein (Cuticle protein)
LAFVEDELEVAEDR	48.2	3.5	1.81E+04	A0A1J1HX79_9DIPT	CLUMA_CG005729, isoform F (tropomyosin 2)
NVVHSDK	31.82	2.6	1.73E+04	E9JC17_SOLIN	Aminopeptidase
LEVSEEK	23.21	4.6	1.73E+04	A0A1J1HX79_9DIPT	CLUMA_CG005729, isoform F (tropomyosin 2)
DLLIACTICGK	24.17	5.8	1.62E+04	B0W4Y6_CULQU	Serendipity locus protein delta
ALGFPFDR	30.41	3.9	1.53E+04	U5EWU6_9DIPT	Putative hexamerin 2 beta
LLAEDADGK	41.32	3.8	1.30E+04	A0A1J1HZZ6_9DIPT	CLUMA_CG005729, isoform B (putative Tropomyosin 2)
LSEASQAADESER	51.57	3.4	1.25E+04	B4QYK2_DROSI	GD19006 (Tropomyosin)
DEEVDEMIR	35.23	4.4	1.18E+04	H9IVN8_BOMMO	Uncharacterized protein (calmodulin)
LVQVEADLVSSK	36.07	3.2	1.16E+04	T1GWE6_MEGSC	Uncharacterized protein (Tropomyosin)
ILEELIEEVDEDK	46.75	3.1	1.15E+04	A0A0L0BTD6_LUCCU	Troponin C, isoform 3
LEDEQSVVGK	29.63	3	1.08E+04	A0A139WE70_TRICA	Myosin heavy chain, muscle-like Protein
VADEYDPHPQY	23.02	3.4	1.08E+04	R4G8D1_RHOPR	Putative cuticle protein
DVQDSLTGDSK	29.68	3.4	1.06E+04	N6UFH9_DENPD	Uncharacterized protein (chitin binding protein)
DVQDSITGDSK	29.68	3.4	1.06E+04	K7IX09_NASVI	Uncharacterized protein (cuticle protein)
AYDVQDSLTGDSK	42.31	4.3	1.06E+04	T1GYP1_MEGSC	Uncharacterized protein (cuticle protein)
AYDVQDSITGDSK	42.31	4.3	1.06E+04	K7IX09_NASVI	Uncharacterized protein (cuticle protein)
HEIASTR	25.31	1.9	1.00E+04	A0A026WSH0_CERBI	Protein scabrous
FRAAVPSGASTGVHEALELR	31.67	4.3	9.60E+03	A0A411G6M9_9HYME	Putative enolase isoform X1

LTQEAVADLER	38.94	3.8	9.59E+03	A0A139WE70_TRICA	Myosin heavy chain, muscle-like Protein
TGYGPLGK	28.6	3.2	9.46E+03	Q0IF51_AEDAE	Protein HIRA
LAMVEADLER	39.22	3.9	8.73E+03	B0X3L6_CULQU	Tropomyosin invertebrate
IEELEEEVEAER	42.35	3.3	7.40E+03	A0A139WE70_TRICA	Myosin heavy chain, muscle-like Protein
AQQELEEAEER	41.11	3.6	6.84E+03	A0A139WE70_TRICA	Myosin heavy chain, muscle-like Protein
HNDAVAEMAEQVDQLNK	28.1	3.5	6.48E+03	W4VRL5_9DIPT	Putative myosin class i heavy chain
ADMAEQAISK	27.42	3.8	6.02E+03	A0A0R1DVF3_DROYA	Myosin heavy chain, isoform D
TGEELQAAEDK	33.08	2.6	4.89E+03	A0A0R1DVF3_DROYA	Myosin heavy chain, isoform D

* GNEFA: guanyl-nucleotide exchange factor activity; NHL: tripartite motif-containing protein 71.

Table S2 Peptide sequences identified in lesser mealworm protein extract by High Resolution Mass Spectrometry on LTQ-Orbitrap instrument. The characterisation reported information about the precision of the MS identification (10lgP and ppm), the peptide abundance (Area), the Uniprot accession number and also the name of the parental protein.

Peptide	-10lgP	ppm	Area	Accession	Protein
SGGTTMYPGIADR	58.22	1	3.79E+06	D6WF19_TRICA	Actin-87E-like Protein
IEELEEEVEAER	60.31	-0.9	3.55E+06	N6T0X7_DENPD	Uncharacterized protein (Myosin)
GIITNWDDMEK	52.01	-0.9	2.97E+06	D6WF19_TRICA	Actin-87E-like Protein
LTQEAVADLER	52.06	-1.1	1.54E+06	N6T0X7_DENPD	Uncharacterized protein (Myosin)
LAEAEETIESLNQK	59.93	0.4	1.53E+06	N6T0X7_DENPD	Uncharacterized protein (Myosin)
AAVPAGSGLEGQWIPDINEK	70.44	-1.1	1.39E+06	Q7M478_TENMO	Cuticle structural protein
TVVADEYDPHPQYS	67.23	-0.5	1.31E+06	R4G8D1_RHOPR	Putative cuticle protein
TTGIVLDSGDGVTHTVPI	42.53	-2.2	1.12E+06	D6WF19_TRICA	Actin-87E-like Protein
GAYEEGQEQLEAVR	62.96	-0.7	1.00E+06	N6T0X7_DENPD	Uncharacterized protein (Myosin)
IEDEIAKLEEK	52.04	-1.3	1.00E+06	N6T0X7_DENPD	Uncharacterized protein (Myosin)
LIDDHFLF	31.04	-1.6	9.32E+05	A0A139WNX9_TRICA	Arginine kinase 1
IVELEEELR	45.47	-0.8	9.32E+05	U4U063_DENPD	Uncharacterized protein (Trpomyosin)
LSIENSDLLR	43.92	-0.5	9.26E+05	N6T0X7_DENPD	Uncharacterized protein (Myosin)
DTQTALEEEQR	49.34	0.7	9.11E+05	A0A139WE70_TRICA	Myosin heavy chain, muscle-like Protein
ELQAALEEAEAALEQEENKVLR	30.12	-0.7	9.01E+05	N6T0X7_DENPD	Uncharacterized protein (Myosin)
GMESCGIHETVY	23.21	-1	8.78E+05	D6WF19_TRICA	Actin-87E-like Protein

MYDGIAELIK	32.32	-0.9	8.66E+05	I4DIQ0_PAPXU	Arginine kinase
SDLESQLSETQDR	62.76	-1.3	8.64E+05	A0A139WE70_TRICA	Myosin
LEDEQSVVGK	51.5	-0.2	8.36E+05	N6T0X7_DENPD	Uncharacterized protein (Myosin)
IADENGFQPH	51.31	-0.5	7.73E+05	U4UAT3_DENPD	Alpha-mannosidase
LFEGGYEEIIK	32.29	2	7.65E+05	V5GZG5_ANOGL	Troponin T
FAYDVQDGLTGDSK	34.45	1	7.34E+05	A0A194QZ16_PAPMA	Cuticle protein
QLEEAESQVNQLSK	61.67	0.9	6.92E+05	V9ICZ0_APICE	Myosin (+ isoform 7)
DLEESNIQHEGTLANLR	68.3	-0.5	6.87E+05	N6T0X7_DENPD	Uncharacterized protein (Myosin)
TVEYTADPVNGF	61.71	0.8	6.49E+05	R4G8D1_RHOPR	Putative cuticle protein
LYDDGSYKPELTPIPL	67.84	0.1	6.44E+05	D6WQN1_TRICA	ADFb like protein
SIIFEDPHPV	38.56	-0.1	6.34E+05	A0A139WGR3_TRICA	Apolipophorins-like protein (lipid transporter activity)
LDEAEANALK	43.86	0.1	6.28E+05	N6T0X7_DENPD	Uncharacterized protein (Myosin)
LLNEDLEIER	44.14	-1.8	6.06E+05	A0A0U2P8E2_9CUCU	Paramyosin
WWNELEAK	28.85	-1.2	5.93E+05	A0A0C5D652_TENMO	Chemosensory protein CSP6 mRNA
KLEADINEL	22.7	-1.1	5.91E+05	N6T0X7_DENPD	Uncharacterized protein (myosin)
AGFAGDDAPR	47.89	-1.2	5.62E+05	D6WF19_TRICA	Actin-87E-like Protein
IINVIGEPIDER	54.51	-0.8	5.54E+05	A0A034VA91_BACDO	ATP synthase subunit beta
VTPEEFVQDSFK	48.97	-1.3	5.48E+05	D6WUQ7_TRICA	Larval serum protein 2-like Protein
YSYETSNGLSSDEQGEVKNEGR	25.26	-0.1	5.08E+05	D6WMB1_TRICA	Larval cuticle protein 8-like Protein
DIEDLELNIQK	53.96	-1.2	5.04E+05	A0A0M4ECL7_DROBS	Myosin
IQEKEEEFENTR	62.14	0.5	4.96E+05	N6T0X7_DENPD	Uncharacterized protein (Myosin)
YKEIGDDLD	37.35	0.1	4.94E+05	U4U063_DENPD	Tropomyosin (+ ISOFORMS C, A, B, G)
HFETSMRDPA	20.02	4.1	4.76E+05	D6WUQ7_TRICA	Larval serum protein 2-like Protein
LELSVPAGSGLEGQWIPDVNEK	66.95	-1	4.68E+05	D6WQM8_TRICA	ADFb like protein
TLNDFDAAK	38.14	-0.9	4.46E+05	N6T0X7_DENPD	Uncharacterized protein (Myosin)
LEEAEGGAESQFEINK	76.89	-1.1	4.36E+05	A0A0U2P8E2_9CUCU	Paramyosin
QLMDHDKDGIITK	32.48	1.4	4.30E+05	D6WZU7_TRICA	Myosin regulatory light chain 2-like Protein
VIQSGLENHDSGIGIYAPDAD	41.28	0.5	4.24E+05	A0A139WNX9_TRICA	Arginine kinase 1
ELEELGER	21.6	0.2	4.10E+05	N6T0X7_DENPD	Uncharacterized protein (myosin)
YFGELNDLR	45.63	-0.3	4.00E+05	N6T0X7_DENPD	Uncharacterized protein (Myosin)
GNLIEGNPDSPYYKYYGAYQVFARHLLG	25.58	-1.4	3.99E+05	D6WUQ7_TRICA	Larval serum protein 2-like Protein

FLAEEADKKYDEVAR	34.2	-0.1	3.99E+05	U4U063_DENPD	Tropomyosin (+ ISOFORMS C, A, B, G)
TIAMDGTEGLVR	62.55	-0.7	3.55E+05	A0A034VA91_BACDO	ATP synthase subunit beta
KFENENIGVDGY	47.57	0.1	3.51E+05	D6WMB2_TRICA	Larval cuticle protein 8-like Protein
TADPIHGF	20.28	0.2	3.48E+05	Q16UU4_AEDAE	AAEL009796-PA (cuticle protein)
ADLAEQAISK	46.09	-1.2	3.46E+05	A0A0M4ECL7_DROBS	Myosin
LQLIEEDLER	38.49	-0.6	3.39E+05	N6UBK5_DENPD	Tropomyosin
IQLLEEDLER	38.49	-0.6	3.39E+05	U4U063_DENPD	Tropomyosin (+ ISOFORMS C, A, B, G)
TVDYTADPHNGF	53.34	-0.3	3.36E+05	A0A194QZ16_PAPMA	Cuticle protein
ANALQNELEESR	50.65	0.3	3.34E+05	N6T0X7_DENPD	Uncharacterized protein (Myosin)
YGPNELPAEEGK	43.61	-1.6	3.30E+05	ATC1_ANOGA	Calcium-transporting ATPase sarcoplasmic/endoplasmic reticulum type (+ ISOFORMS A,B)
DLQIEVDR	25.89	-1.3	3.28E+05	A0A139WE70_TRICA	Myosin
DLQLEVDR	25.89	-1.3	3.28E+05	N6T0X7_DENPD	Uncharacterized protein (Myosin)
EAALEQEENKVLR	30.55	0	3.27E+05	N6T0X7_DENPD	Uncharacterized protein (Myosin)
IEDLELNIQK	31.95	-0.2	3.22E+05	A0A0M4ECL7_DROBS	Myosin
MVGPIEEVVQK	51.09	-1.6	3.15E+05	A0A034VA91_BACDO	ATP synthase subunit beta
IMELEEELK	30.74	0	3.02E+05	A0A0A1E5I3_MONAT	Tropomyosin (isoform 1)
KQEGEDPDPTPY	37.14	0.7	3.01E+05	A0A1L8E579_9DIPT	Myosin
TEMSETEEIKTPL	46.71	-0.4	2.95E+05	ATC1_ANOGA	Calcium-transporting ATPase sarcoplasmic/endoplasmic reticulum type (+ ISOFORMS A,B)
GEYIGDGDYHGEGLAEA	62.36	-0.3	2.89E+05	D6WT50_TRICA	structural constituent of cuticle
LSILEEESMFPK	58.64	-1.2	2.77E+05	N6T0X7_DENPD	Uncharacterized protein (Myosin)
MDALENQLK	33.38	0.8	2.69E+05	U4U063_DENPD	Tropomyosin (+ ISOFORMS C, A, B, G)
TVEYTADPINGF	40.66	-0.3	2.68E+05	CUA1A_TENMO	Larval cuticle protein A1A
TVVADEYDPNPQYSFGYDVQD	63.74	-0.7	2.62E+05	CUA1A_TENMO	Larval cuticle protein A1A
IMDPNIIGQEHY	41.78	0.2	2.55E+05	A0A034VA91_BACDO	ATP synthase subunit beta
KITDVGDVVVD	25.22	-0.9	2.53E+05	A0A1B6E9S2_9HEMI	Calcium-transporting ATPase
FDRPVY	30.59	0.4	2.47E+05	D6WUQ7_TRICA	Larval serum protein 2-like Protein
ETGNGIAAQEQGQLK	53.42	-0.9	2.42E+05	A0A139WHF4_TRICA	Chitin binding protein

IGVFGEDEDTTGK	55.84	-3.1	2.39E+05	ATC1_ANOGA	Calcium-transporting ATPase sarcoplasmic/endoplasmic reticulum type (+ ISOFORMS A,B)
LLAEDADNKSDEVSR	61.95	-0.7	2.32E+05	A0A0A1E5I3_MONAT	Tropomyosin (isoform 1)
VRELENELD	20.92	0.3	2.29E+05	N6T0X7_DENPD	Uncharacterized protein (myosin)
SLVDPDGTR	29.75	-0.7	2.19E+05	CUA1A_TENMO	Larval cuticle protein A1A
VSSTLSGLEGELK	39.65	1.8	2.16E+05	A0A139WNX9_TRICA	Arginine kinase 1
LEEVASKF	28.62	0	2.01E+05	D5LG83_LYSTE	Arginine kinase
VEEGAGDPEFIKR	46.99	0.2	2.00E+05	D6W953_TRICA	Troponin T
IDQSILTGESVSVIK	61.36	0.1	1.99E+05	ATC1_ANOGA	Calcium-transporting ATPase sarcoplasmic/endoplasmic reticulum type (+ ISOFORMS A,B)
QEQEVNFDGSYH	39.81	0.2	1.81E+05	A0A139WHF4_TRICA	Chitin binding protein
WMSGEEFNLK	27.57	-0.2	1.72E+05	D6WUQ7_TRICA	Larval serum protein 2-like Protein
VAVADEYDPHPQYS	43.47	-1.3	1.61E+05	D6W8Q5_TRICA	Pupal cuticle protein
NLNDEIAHQDELINKL	23.97	-3.3	1.59E+05	N6T0X7_DENPD	Uncharacterized protein (myosin)
YPDVHELAK	30.88	0.7	1.42E+05	tr|A0A1A9YR78|A0A1A9YR78_GLOFF	Phosphofructokinase
SDLDEQLR	41.59	0.5	1.42E+05	V5GZG5_ANOGL	Troponin T
LKVDDLAAELD	37.21	-0.8	1.36E+05	N6T0X7_DENPD	Uncharacterized protein (Myosin)
SYELPDGQVITIGNER	53.6	-2.1	1.32E+05	D6WF19_TRICA	Actin-87E-like Protein
AQQELEEAEER	47.71	-0.7	1.30E+05	N6T0X7_DENPD	Uncharacterized protein (Myosin)
FTEEQLR	35.57	-0.1	1.22E+05	C9X4E7_9CUCU	Muscular protein 20
GAPEGVLER	26.1	-0.6	1.17E+05	ATC1_ANOGA	Calcium-transporting ATPase sarcoplasmic/endoplasmic reticulum type (+ ISOFORMS A,B)
LEEAGGATSAQIELNK	62.83	-1.6	1.14E+05	N6T0X7_DENPD	Uncharacterized protein (Myosin)
IQEKDEEIEAIRK	22.84	-1.7	1.14E+05	A0A0U2P8E2_9CUCU	Paramyosin
LQEKDEEIEAIRK	22.84	-1.7	1.14E+05	E0W1N5_PEDHC	Paramyosin, long form, putative
FGYDVQDGLTGDSK	65.1	1.6	1.04E+05	CUA1A_TENMO	Larval cuticle protein A1A
NALEQANKDLEEKEK	22.14	0.3	1.04E+05	A0A1J1HVX0_9DIPT	Uncharacterized protein (myosin)
FETSDPISR	40.49	0.1	1.02E+05	D6WMB3_TRICA	Larval cuticle protein
GFTQEEKDNIYK	35.93	1	9.97E+04	Q178Y3_AEDAE	Actin filament binding

AGVLGQMEELR	40.44	-0.2	9.31E+04	N6T0X7_DENPD	Uncharacterized protein (Myosin)
HVGDLGNVEAGGDGVAK	66.17	-0.6	9.13E+04	A0A076G467_TENMO	Superoxide dismutase [Cu-Zn]
DGPQAINNQGGAPNYHPN	52.77	0.1	8.82E+04	A0A139WLA9_TRICA	Catalase
IGELENQKY	28	-0.5	8.76E+04	A0A139WJG4_TRICA	Troponin
SSLEGEKGSLSEVQER	44.97	-1.1	8.68E+04	A0A139WE70_TRICA	Myosin
QLQEQEGMSQQNVTR	57.56	2	8.57E+04	E0W1N5_PEDHC	Paramyosin
SNDIHDYY	32.31	-0.7	8.49E+04	A0A1L8E579_9DIPT	Myosin
PEEHPVL	20.89	-0.4	8.17E+04	D6WF19_TRICA	Actin-87E-like Protein
KHNDAVSEMGEQLD	51.04	-0.4	7.52E+04	H9JXG1_BOMMO	Myosin
KHNDAVSEMGEQID	51.04	-0.4	7.52E+04	N6T0X7_DENPD	Uncharacterized protein (Myosin)
VIVITGDNK	38.26	-1.2	7.40E+04	ATC1_ANOGA	Calcium-transporting ATPase sarcoplasmic/endoplasmic reticulum type (+ ISOFORMS A,B)
GMDFQPR	24.76	1.6	6.94E+04	D2A424_TRICA	Phenoloxidase subunit A3-like Protein
DGDVVHGSY	47.46	0	6.90E+04	R4G8D1_RHOPR	Putative cuticle protein
IWIDGTGEYVR	42.53	-1.9	6.74E+04	A0A067R5C5_ZOONE	Glutamine synthetase
LAEASQAADESER	67.79	-0.2	6.66E+04	U4U063_DENPD	Tropomyosin (+ ISOFORMS C, A, B, G)
KSFDENGTIDSER	28.77	0.8	6.60E+04	D6WZU7_TRICA	Myosin regulatory light chain 2-like Protein
AADFIQER	32.91	-1.3	6.20E+04	A0A139WLA9_TRICA	Catalase
STAGDTHLGGEDFDNR	60.04	-0.1	5.37E+04	I6SMI7_BICAN	Heat shock cognate 70
LGPKYDEYGR	23.52	-2.9	4.96E+04	D6WUQ7_TRICA	Larval serum protein 2-like Protein
AQIPAGVDAR	26.3	-1.1	4.94E+04	D6WT50_TRICA	structural constituent of cuticle
SQNSFLPR	29.87	-0.4	4.55E+04	A0A0L7LKM3_9NEOP	Apolipophorin protein
FENVKESKNI	21.07	-1.3	3.94E+04	Q6NL43_DROME	GM10157p (Zinc finger_CCHC)
DNAQDKADAMEGQAK	26.38	-0.9	3.39E+04	A0A139WAP9_TRICA	Tropomyosin (Isoform 2)
GEYSYVGPDGK	25.42	-2.3	2.94E+04	D6WMB2_TRICA	Larval cuticle protein 8-like Protein
LIAEDVQGR	35.12	-1.9	2.93E+04	A0A0L7KSL2_9NEOP	40S ribosomal protein S3a
LNSQVNDLR	24.46	-2.7	2.90E+04	A0A139WJG4_TRICA	Uncharacterized protein (troponin)
KQEYDESGPGIVHR	31.3	0.5	2.69E+04	D6WF19_TRICA	Actin-87E-like Protein

Additional material to the gel electrophoresis analysis

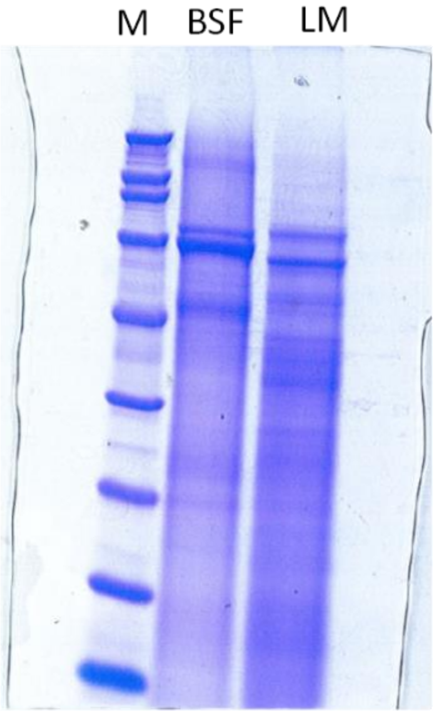

Figure S1 Full-length SDS-Page of black soldier fly (BSF) and lesser mealworm (LM) larvae

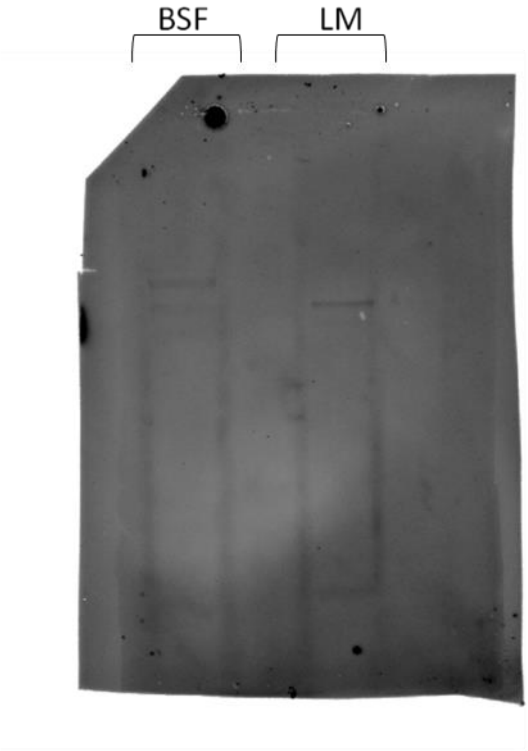

Figure S2 Full-length IgG-immunoblotting of the samples separated by SDS-Page (Figure S1), followed by incubation with anti-tropomyosin I antibody. BSF: black soldier fly; LM: lesser mealworm.

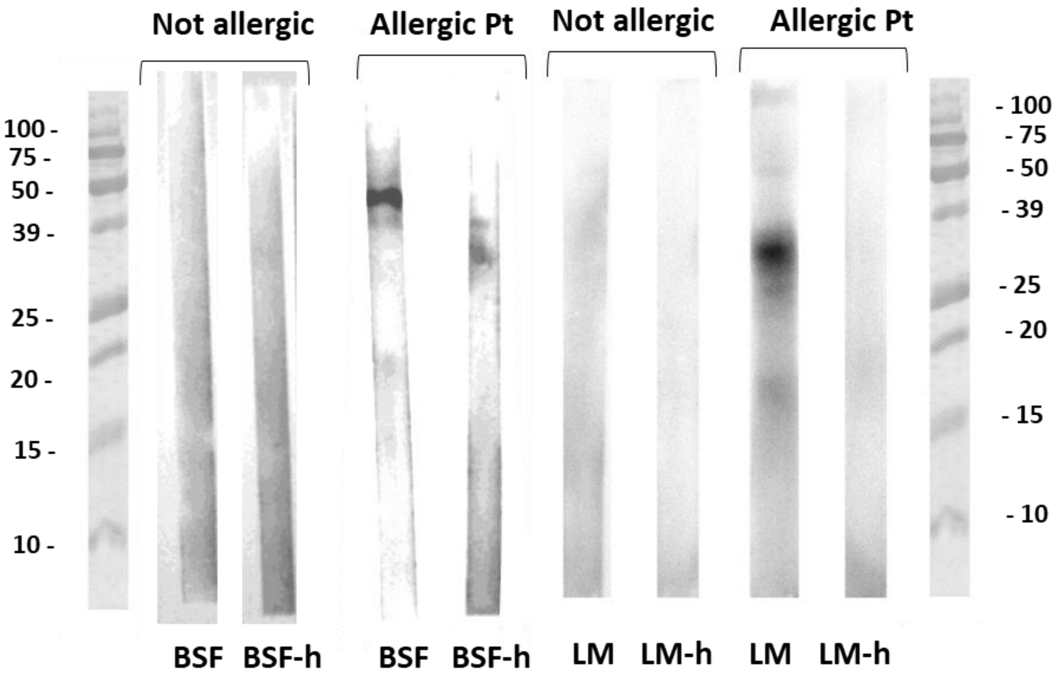

Figure S3 Full-length IgE-Immunoblotting results after incubation with sera from non-allergic patient (Pt) and person allergic to crustacean tropomyosin. BSF: black soldier fly; LM: lesser mealworm; h: hydrolysate.

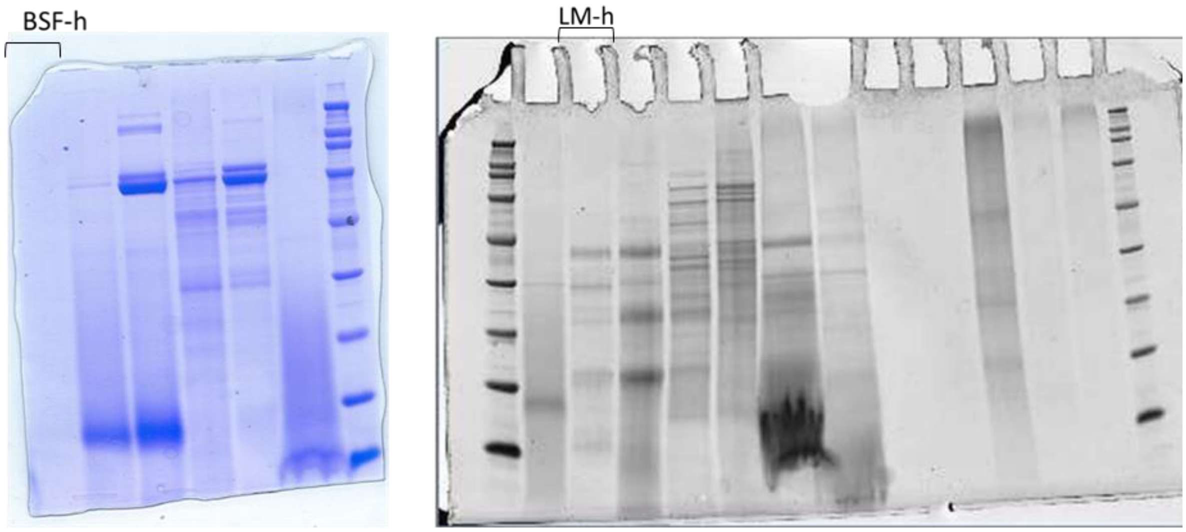

Figure S4 and S5 Full-length SDS-Page of protein hydrolysates obtained from proteolysis activity of the protease from *Bacillus licheniformis* on black soldier fly (BSF-h) and lesser mealworm (LM-h) protein hydrolysates

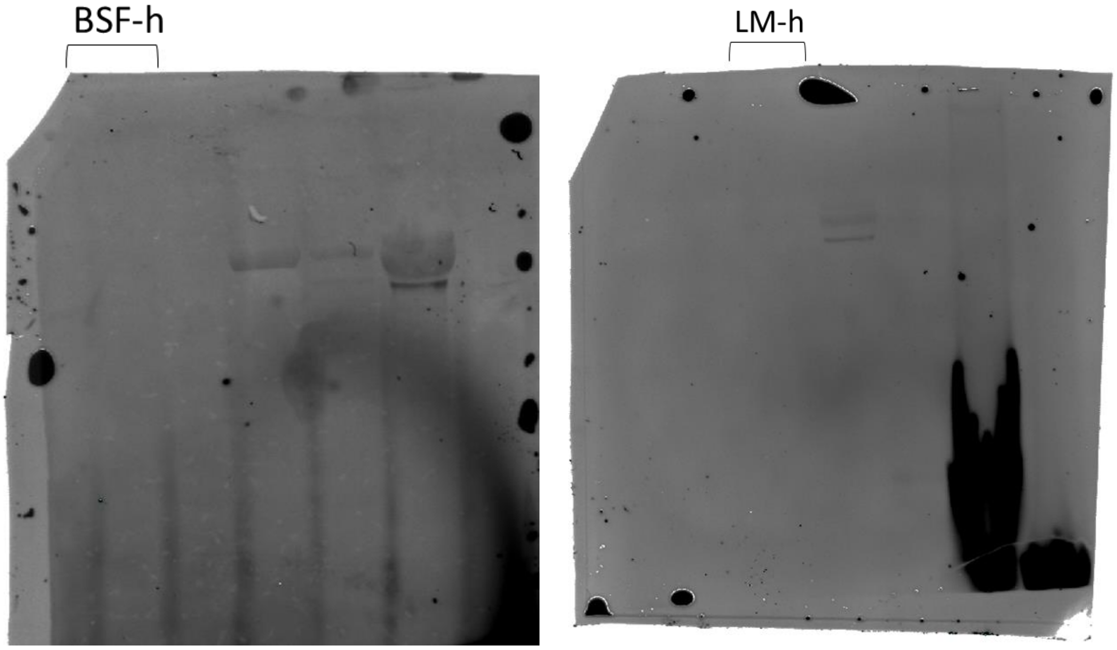

Figure S6 and S7 Full-length IgG-immunoblotting of the samples separated by SDS-Page, followed by incubation with anti-tropomyosin I antibody. BSF-h: black soldier fly hydrolysate; LM-h: lesser mealworm hydrolysate.
